# Costs and mortality rates of surgical approaches to hysterectomy in Brazil

**DOI:** 10.11606/S1518-8787.2018052000129

**Published:** 2018-03-12

**Authors:** Kathiane Lustosa Augusto, Aline Veras Morais Brilhante, Gisele Cristine Duarte Modesto, Dayana Maia Saboia, Cássia Fernandes Coelho Rocha, Sara Arcanjo Lino Karbage, Thaís Fontes de Magalhães, Leonardo Robson Pinheiro Sobreira Bezerra

**Affiliations:** I Universidade Federal do Ceará. Faculdade de Medicina. Maternidade Escola Assis Chateaubriand. Fortaleza, CE, Brasil; II Universidade de Fortaleza. Programa de Pós-Graduação em Saúde Coletiva. Fortaleza, CE, Brasil; III Maternidade Escola Assis Chateaubriand. Universidade Federal do Ceará. Fortaleza, CE, Brasil; IV Universidade Federal do Ceará. Programa de Pós-Graduação em Enfermagem. Fortaleza, CE, Brasil; V Universidade Federal do Ceará. Faculdade de Medicina. Maternidade Escola Assis Chateaubriand. Fortaleza, CE, Brasil; VI Universidade Federal do Ceará. Programa de Pós-Graduação em Ciências Clínico-Cirúrgicas. Fortaleza, CE, Brasil

**Keywords:** Hysterectomy, economics, Hysterectomy, mortality, Health Care Costs, Neoplasms, prevention & control, Women's Health Services, Public Health

## Abstract

**OBJECTIVE:**

To analyze the costs of hysterectomies performed in Brazil due to benign conditions, and to assess its hospital admittance and mortality rates.

**METHODS:**

A retrospective cohort was carried out from January 2010 to December 2014, analyzing all hysterectomies (n = 428,346) registered on the DATASUS database between January 2010 and December 2014. Data were collected through a structured questionnaire and analyzed using the SPSS 20.0 for Windows.

**RESULTS:**

Hospital admissions were 300,231 for total abdominal hysterectomies, 46,056 for vaginal hysterectomies, 29,959 for subtotal abdominal hysterectomies and 1,522 for laparoscopic hysterectomies. Mortality rates were 0.26%, 0.09%, 0.07% and 0.05% for subtotal, total abdominal, laparoscopic, and vaginal hysterectomies, respectively. Among the procedures studied, total abdominal hysterectomies had the most costs (R$217,802,574.77), followed by vaginal hysterectomies (R$24,173,490.00), subtotal abdominal hysterectomies (R$19.253.300,00) and laparoscopic hysterectomies (R$794,680.40).

**CONCLUSIONS:**

Total abdominal hysterectomies had the highest overall costs mainly because it was the most commonly performed technique. Mortality rates were greatest in subtotal abdominal hysterectomies; this, however, may be due to bias related to missing data in our database.

## INTRODUCTION

Technological advancements in healthcare have been associated with increasing expenses, thus raising the need for cost control strategies^3^. The first step in this process is to analyze the overall cost of medical procedures, as well as their cost-benefit and cost-effectiveness, aiming for the best patient care and smallest impact in economic resources^6^. In the Brazilian public health system (SUS), hysterectomies are the second most common surgery performed in women of reproductive age, the first being the cesarean delivery. Therefore, a study comparing cost-benefit between different hysterectomy techniques is of interest to public health and healthcare budget management.

Hysterectomies can be done transabdominally (through laparotomy), transvaginally, or in a minimally invasive fashion (with or without robotic assistance)^8^. Abdominal hysterectomy (AH) refers to the removal of the uterus through an incision in the inferior abdomen, and may be total (if the uterus is removed in its entirety) or subtotal (if the uterine cervix is spared). Vaginal hysterectomy refers to the removal of the uterus through the vagina, is always total, and does not require any abdominal incisions^14^.

Three approaches to hysterectomy for benign diseases are possible: AH, vaginal hysterectomy (VH), and laparoscopic hysterectomy (LH). The LH, in turn, has three further subdivisions: laparoscopic-assisted vaginal hysterectomy (LAVH), when the uterine removal is assisted by laparoscopic procedures that do not include uterine artery ligation; laparoscopic hysterectomy (LH), when the laparoscopic procedures include uterine artery ligation; and total laparoscopic hysterectomy (TLH), when there is no vaginal component and the vaginal cuff is sutured laparoscopically^17^.

In order to improve patient outcomes, minimally invasive surgical techniques have been developed, resulting in smaller incisions, less post-operative pain, faster recovery and return to baseline activities, and decreased surgical morbidity^8^. This group includes TLH, which is the detachment of the entire uterine cervix and body via the laparoscope; LAVH, when the removal of the uterus is completed through the vagina along with ligation of the cardinal ligament and suture of the vaginal cuff^13^; and robotic-assisted (RA) procedures, which facilitate the use of laparoscopes by allowing increased precision and ergonomics^7^. Although AH and VH remain the most commonly performed techniques, there has been an increase in the frequency of minimally invasive techniques in the past few years^2^.

Nonetheless, the choice of hysterectomy technique to be employed does not depend solely on surgical costs. Among other factors, accounting for its related morbimortality is crucial. With this in mind, this study aimed to assess the costs and mortality of surgical techniques for hysterectomies performed in Brazil, and to compare such techniques regarding public costs, mortality and hospital admission rates.

## METHODS

This retrospective cohort study was carried out from January 2010 to December 2014, analyzing all hysterectomies (n = 1,132,123), and analyzed all hysterectomies (abdominal, vaginal and laparoscopic) due to benign conditions performed in the Brazilian Public Health System and registered on the DATASUS database between January 2010 and December 2014.

Surgical instruments, room, and personnel cost were included as costs. Such costs include only those dispensed by the healthcare provider, that is, the Brazilian healthcare system. Out-of-pocket expenses for patients and their families were not considered.

Statistical analyses were carried out for the following categories: total procedure costs, procedure-related mortality rate, number of deaths, and number of hospital admissions. Mean and standard deviation (SD) or median and 95% confidence interval (CI) were calculated.

Possible associations between independent variables (LH, VH, SAH, TAH, number of hospital admissions and daily costs related to the procedure) and outcome variables (total costs of the procedure, procedure-related mortality rate, number of deaths, and number of hospital admissions) were tested. Data were also statistically analyzed in subdivisions according to geographic regions. For all tests, a p-value lesser than 5% (p < 0.05) was considered significant.

Considering the asymmetrical distribution of costs data, the nonparametric, Kruskal-Wallis test was used from SPSS 2015. The research that originated this article was carried out with a search in the database DATASUS, a public domain database. It was not characterized as a research involving human beings and does not need approval by an ethics committee.

## RESULTS

A total of 428,346 hospital admissions due to benign hysterectomies were reported for the period studied. The most commonly used technique was open surgery (88%), followed by vaginal (12%) and laparoscopic (0.35%) approaches.

**Figure 1:**
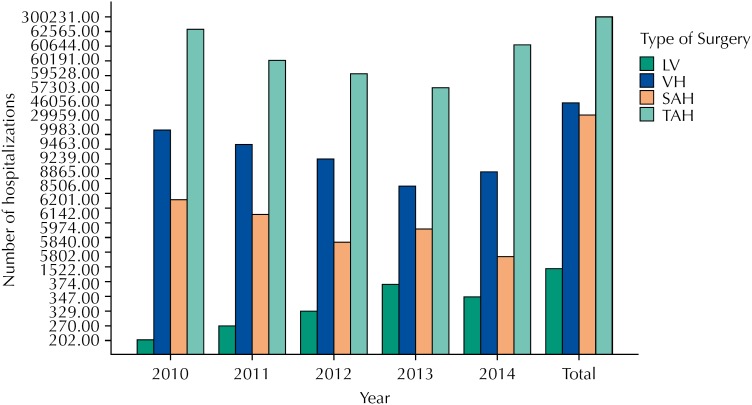
Yearly number of hospital admissions related to each type of hysterectomy. LV: videolaparoscopic hysterectomy VH: vaginal hysterectomies; SAH: subtotal abdominal hysterectomies; TAH: total abdominal hysterectomies

**Figure 2:**
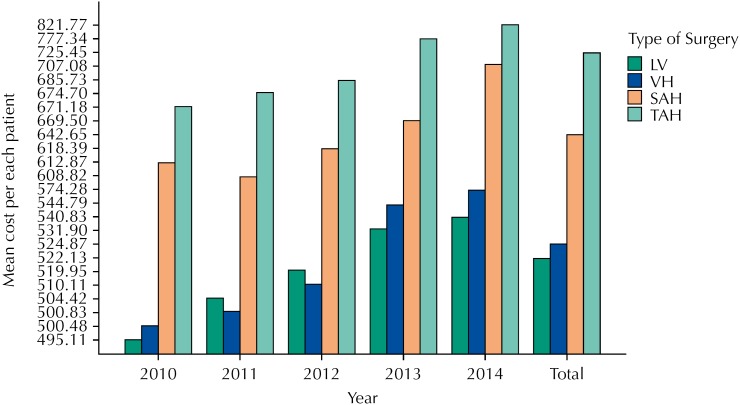
Mean cost per each patient that underwent each surgical approach, shown by year. LV: videolaparoscopic hysterectomy; VH: vaginal hysterectomies; SAH: subtotal abdominal hysterectomies; TAH: total abdominal hysterectomies

Despite the predominance of TAH seen in Figure 1, Figure 2 illustrates the increase in the number of laparoscopic procedures and the decrease in the proportion of TAH and VH performed in 2010-2013. Interestingly, in 2014, there was a slight increase in the latter two approaches and a decrease in the number of LH, when compared to the previous year.

The mean cost, per patient, for each type of surgical technique considering all years studied were R$725.45, R$642.65, R$524.87 and R$522.13 for TAH, SAH, VH and LH, respectively. This data is shown in Figure 2.

**Figure 3:**
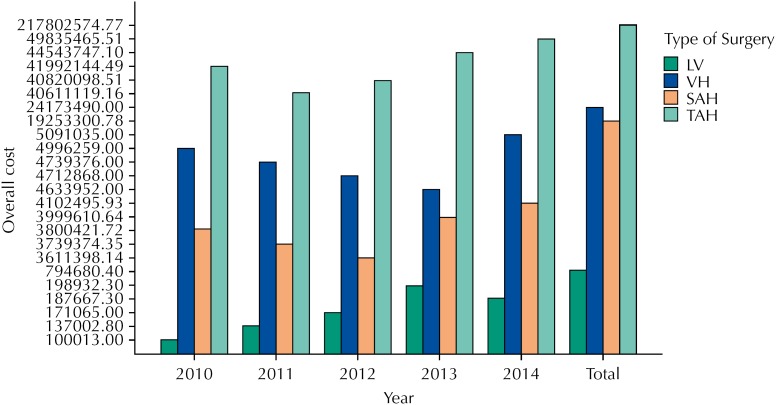
Overall costs for each surgical technique, shown per year. LV: videolaparoscopic hysterectomy VH: vaginal hysterectomies; SAH: subtotal abdominal hysterectomies; TAH: total abdominal hysterectomies

It is clear, thus, that the technique with the highest associated costs to public health was TAH, not only for being the most commonly performed procedure but also for carrying the highest cost per patient. The expenses with this type of procedure added up to R$217,802,574.77 in this five-year period. Figure 3 illustrates the sum of costs for each surgical approach, shown by year.

There were no statistically significant differences between each surgical technique regarding mortality rates. The rates found for each approach between 2010-2014 were 0.06%, 0.07%, 0.09% and 0.26% for VH, LH, TAH and SAH, respectively.

After the evaluation of the data by Kruskal-Wallis test demonstrated that, in general, Brazil had no significance in relation to the cost per day of hospitalization and mortality rates for all types of hysterectomies performed in the evaluated period.

The comparison between surgical techniques demonstrated, in relation to vaginal hysterectomy compared to total abdominal hysterectomy (TAH) and abdominal subtotal (SAH), that the cost per day of hospitalization was lower vaginally (p = 0,042). The cost per day of hospitalization for laparoscopic hysterectomy was less than total hysterectomy, and subtotal abdominal had lower costs than total abdominal (p < 0,001).

Regarding the North of Brazil, there was no significant difference in cost per day of hospitalization among the techniques used, but it was between VH and TAH (p < 0,001). There was no significant difference in relation to mortality among the techniques.

In the Northeast, there was a significant difference between VH and TAH (p = 0,004) and between LH and TAH (p = 0,017). There was no significant difference in the daily cost among the other surgical techniques performed in general and in mortality rate.

The Southeast region showed a difference in the cost of hospitalization per day and the mortality rate of hysterectomies performed in general. Comparing the techniques together, in relation to videolaparoscopic hysterectomy and vaginal compared with abdominal total (p = 0,005 and p = 0,033), the first had lower costs per day of hospitalization. The mortality rate was higher when comparing abdominal total and vaginal (p = 0,012), there was no difference with the other techniques in this region.

In Midwestern Brazil, there was no difference between the rate of mortality and the cost per day of hospitalization among the surgical techniques performed.

By comparing each surgical technique among the regions of Brazil, it showed a significant difference in hospitalization costs in hysterectomies types, total abdominal and abdominal subtotal except in videolaparoscopic. The mortality rate overall was higher in TAH, there was no significance in the vaginal and subtotal, and videolaparoscopic could not be calculated due to lack of data.

## DISCUSSION

This study shows substantially reduced costs for LH and VH over other hysterectomy techniques. The TAH was the most expensive approach, with the greatest financial burden to the hospital. These findings corroborate the literature regarding VH, but diverge on the costs of TAH and LH.

Lumsden et al.^11^ found that open abdominal hysterectomy is less expensive than LAVH. It might be worth noting that, in the Lumsden study, half of the patient cohort was lost to follow-up. In the Evaluate hysterectomy trial, a major multicenter randomized-controlled trial, Garry et al.^4^ compared abdominal, vaginal and laparoscopic hysterectomies, and concluded that LH was not cost-effective relative to VH. The Evaluate study^4^ also found that LH was more expensive than AH. According to Dayaratna^3^, VH was the only minimally invasive type of hysterectomy that generated net hospital income. Authors found that hospital costs were larger in LH and RA hysterectomy than in HV Sculpher et al.^15^ also described LAVH as unlikely to be as cost-effective as VH, reporting a mean cost £401 higher than the latter.

However, Warren et al.^16^ found that AH and LH showed comparable costs, with patients with AH presenting longer hospital stays, and conclude that the best cost-effectiveness is seen in VH. In part, these findings are explained by the fact that, depending on the type of disposable equipment used, VH generally requires no additional specific materials when compared to LH. Hence, the cost of LH is usually significantly higher than that of VH^5^.

Several studies have reported no significant differences in outcomes between the different techniques of hysterectomy^4^ ^,^ ^5^ ^,^ ^10^ ^,^ ^15^ ^,^ ^16^, which corroborates the lack of significant differences in mortality rates described in our study.

A systematic review published in the Cochrane Database in 2009 supports the opinion that, when feasible, VH is the safest and the most cost-effective route to remove the uterus^12^. As a primary route of hysterectomy, VH is the method recommended by the American College of Obstetricians and Gynecologists^1^ and has been shown to be less costly than either abdominal or traditional laparoscopic approaches.

Despite such evidence, abdominal hysterectomy is still the most commonly used technique. The decrease of hysterectomy rates seen over the years is consistent with the findings reported by Jonsdottir^9^, who found that the frequency of AH decreased significantly from 2006 (64.7%) to 2009 (35.8%). The proportion of VH did not change significantly, but the percentage of laparoscopic cases increased from 17.7% in 2006 to 46% in 2009. In our data, however, the decrease in TAH was accompanied by a decrease in VH as well.

The total cost of laparoscopic hysterectomy and vaginal were lower when compared to other techniques and in all regions of Brazil. The mortality rate was higher when comparing abdominal total and vaginal, there was no difference with the other techniques both in the South and in the Southeast. In other regions, we could not compare for lack of reliable data. The TAH was the technique carrying the highest cost per patient. Nevertheless, this was the surgical technique most used. This finding raises important questions for public health and development control strategies.
